# Exploring the performance of volatile mutations on evolutionary game dynamics in complex networks

**DOI:** 10.1016/j.heliyon.2023.e16790

**Published:** 2023-06-02

**Authors:** K.M. Ariful Kabir, MD Shahidul Islam, Sabawatara Nijhum

**Affiliations:** a Department of Mathematics, Bangladesh University of Engineering and Technology, Dhaka, 1000, Bangladesh; b Department of Computer Science and Engineering, Green University of Bangladesh, Dhaka, Bangladesh; c Independent University, Bangladesh, Dhaka, Bangladesh

**Keywords:** Evolutionary game theory, Volatile mutation, Non-linear dynamics, Pairwise game

## Abstract

The typical framework of replicator dynamics in evolutionary game theory assumes that all mutations are equally likely, meaning that the mutation of an evolving inhabitant only contributes constantly. However, in natural systems in biological and social sciences, mutations can arise due to their repetitive regeneration. The phenomenon of changing strategies (updating), typically prolonged sequences repeated many times, is defined as a volatile mutation that has been overlooked in evolutionary game theory. Implementing a repeated time framework introduces a dynamic mutation aspect incorporated with the pairwise Fermi rule. Network structure, ubiquitous in many natural and artificial systems, has significantly affected the dynamics and outcomes of evolutionary games. We examine the evolution of the pairwise game in terms of dilemma strength. It is revealed that mutation intensity can influence evolutionary dynamics. We also demonstrated that the obtained outcomes run by the deterministic and multi-agent simulation (MAS) process present similar stability regions for both linear and non-linear dynamics, even in various game classes. In particular, the most stimulating effect is detected for the relationship between the fraction of cooperation and the fraction of the mutated individuals, as inclination tends to provide an increasing tendency and supporting defection in the opposite case. In conclusion, we identified a form of volatile mutation as a form of noise that, under certain situations, could be used to enhance cooperation in social systems and design strategies for promoting cooperation in networked environments.

## 1 Introduction

In evolutionary game dynamics [[1], [2], [3], [4]], which examine how phenotypes evolve, researchers typically analyze the results of a population of strategies playing a game under the influence of selection and mutation [[5], [6], [7]]. The replicator equation represents a system of differential equations that describes how the frequencies of different strategies evolve, considering both selection and mutation processes [8]. Until now, all researchers [[9], [10], [11], [12], [13], [14]] have studied static or constant mutation by considering linear dynamics in the replicator-mutator equation. Besides, the present study aims to establish a volatile mutation approach on a pairwise replicator dynamic model encompassing linear and non-linear strategy-updating dynamics, where mutation changes at the end of each season. This analysis also includes the bidirectional mutation that mainly incorporated examples from the evolutionary game theory of pairwise two-by-two games.

Dyadic (pairwise) games hold particular significance in evolutionary game theory as they capture the evolving interactions between individuals based on the payoff outcomes of their behaviors. Let’s consider the interaction between two strategies, C and D, within an evolutionary game theory framework with four potential gains. Here, a player receives a reward (R) when both cooperate and incurs a punishment (P) when both players defect. Additionally, there are two payoffs: sucker (S) when one player cooperates and the other defects, and temptation (T) when one player defects while the other cooperates [15,16]. The concept of universal dilemma strength (SD) [[17], [18], [19], [20], [21]] considers two parameters: $D_{g}^{\prime }$ and $D_{r}^{\prime }$, that determine the level of social viscosity required or the degree to which anonymity should be reduced to mitigate social dilemmas across different game classes. These game classes include the Prisoner's Dilemma (PD), Chicken (CH) game, Stag Hunt (SH), and Trivial game (TR), each characterized by distinct behavioral dynamics by, $D_{g}^{\prime }>0$ & $D_{r}^{\prime }>0$, $D_{g}^{\prime }>0$ & $D_{r}^{\prime }<0$ , $D_{g}^{\prime }<0$ & $D_{r}^{\prime }>0$ , and $D_{g}^{\prime }<0$ & $D_{r}^{\prime }<0$. To implement the dynamic mutation effect in the dyadic game, we consider a volatile mutation process on the repeated seasons instead of relying on the constant mutation rate. Furthermore, the dynamic mutation will analyze the cyclic three-strategy dominance game by considering linear and non-linear replicator dynamics [[22], [23], [24], [25], [26], [27]] over a three-strategy game of inter-and intraspecific competitions. In the context of EGT, a commonly made assumption is known as constant mutation, which refers to the fitness mechanism of a particular strategy update process independent of the mutation feedback on the population dynamics. This assumption implies that the fitness mechanism for updating a particular strategy remains constant, regardless of the feedback from mutations on population dynamics. This assumption is often applied in the analysis of short-term evolution, where subtle changes may not be detectable, and the influence of mutation on population dynamics needs to be addressed. Imhof et al. [28] explore the concept of local mutation in the continuous strategy space, specifically focusing on direct reciprocity. Fudenberg et al. [29] investigate pairwise games in finite populations, considering small mutations and non-weak selection scenarios. Binmore et al. [30] and Willensdorfer et al. [31] examine the impact of different mutation rates in the context of resource games (non-generic games) and average population fitness, respectively. Antal et al. [32] analyze the mutation-selection dynamics in pairwise games with multiple strategies using the Moran process within well-mixed populations. Perc [33] conducted a study to analyze how introducing random variations in space and time affects the spatial prisoner’s dilemma game. Helbing et al. [34] examined how effective costly punishment is at promoting cooperation and considered the impact of strategy mutations in addition to the typical dynamics of strategy adoption. Besides, several works aim to deepen our understanding of the behavior of evolutionary games in the presence of stochasticity and mutations. For example, an investigation conducted by Hong Duong and Han [35] focused on analyzing the statistical properties of equilibria in random social dilemma games involving mutation. In a separate study, Duong and Han [36] explored the equilibrium properties of the replicator-mutator equation within deterministic and random game settings. Finally, Chen et L [37]. centered around the number of equilibria in the replicator-mutator dynamics for noisy social dilemmas. Additionally, Toupo and Strogatz [38], Mobilia [39], Hu et al. [40], and Nagatani et al. [41] explore the idea of different mutations as a form of bidirectional mutation, limit-cycle oscillation, imitative dynamics, and paradoxical effect on metapopulation RPS model. Recently, Kabir et al. [27] provided a general analysis of bidirectional mutation for the symmetric rock-paper-scissors game as a noise. None of these works mentioned above further detail how the volatile mutation could change over a generation on the global time scale. Their approach mainly focuses on deterministic and linear dynamics in an infinite population with constant mutation rates. Here, we introduce a volatile mutation equation that eventually leads the system to follow environmental feedback, depending on cooperation and defection present in the system. Further, Replicator-mutator dynamics [36,37] and mutation over a generation are two concepts in evolutionary game theory that explain how populations and strategies evolve. Replicator-mutator dynamics refers to how successful strategies are replicated with occasional mutations that can lead to novel adaptations and behaviors. This explains the emergence of cooperation and altruism in some situations. Mutation over generation refers to how genetic mutations introduce random variations that can change the players' behavior, leading to the evolution of new, more effective strategies through natural selection. High mutation rates can increase variation and the potential for novel strategies but also increase the likelihood of harmful mutations.

Exploring evolutionary game theory has proven to be a valuable approach to comprehending how cooperation arises and persists in social systems. Recently, there has been an increasing focus on investigating the effects of volatile mutation, which introduces stochasticity into the evolutionary process, on the dynamics of evolutionary game theory within network structures. A pairwise two-by-two game on networks with the mutation has been a topic of interest for a more realistic representation of interactions in natural and social systems. The classic evolutionary game theory model assumes that individuals cannot change their strategies after adopting them. However, mutation is a natural mechanism that can create new strategies and eliminate others. Several studies have examined the influence of mutation on pairwise interactions. For instance, Van Segbroeck et al. [42] researched the role of mutation in the evolution of cooperation within spatially structured populations. Their findings revealed that, under certain circumstances, the mutation could promote cooperation’s emergence and sustainability. Another investigation by Su and Li [43] focused on studying the effects of mutation in the evolution of public goods games within scale-free networks. They showed that mutation could promote the emergence of cooperative behaviors under certain conditions. These studies highlight the importance of considering mutation when studying evolutionary game dynamics on networks.

Upon thoroughly exploring and introducing the proposed model, the subsequent section is dedicated to presenting the outcomes and discussing the results obtained from numerical simulations. Finally, we discuss the conclusions drawn from the study and delve into the potential implications stemming from these findings.

## 2 Model and method

Replicator dynamics is a fundamental aspect of evolutionary games, involving individuals who interact with their neighbors and change their strategies based on accumulated payoffs. The strategy update rules can be specified in various ways, such as linear or non-linear dynamics.

A general aspect of evolutionary games is conveyed by the strategy update rules of replicator dynamics, in which individuals interact with their neighbors accumulating a payoff and changing strategy. Such rules can be specified by considering several aspects, such as linear and non-linear dynamics.

### 2.1 Linear dynamics

Consider the general case of multi-strategy with multiplayer games with the set of replicator equations considering bidirectional mutation:

(1.1) $${\dot{x}}_{i}=x_{i}\left[\pi_{i}-\left\langle \pi \right\rangle \right]-\mu x_{i}+\frac{\mu }{n-1}\sum_{j\neq i}x_{i}$$

(1.2) $$\pi_{i}=\sum_{j}x_{j}a_{ij}$$

(1.3) $$\left\langle \pi \right\rangle =\sum_{i}x_{i}\pi_{i}$$

In a population of n types (equations (1.1), (1.3)), the frequencies of type i can be denoted as $x_{i}$, and their fitness as $\pi_{i}$, where i=1,2,3,……n and $\sum_{i=1}^{n}x_{i}=1$. The entities $a_{ij}$ characterize the interactive payoff outcomes of the species. Here, the bidirectional mutation rate is μ, which defines a concurrent biological and ecological activity providing the factors on which evolutionary forces can act. The result of each contribution is a linear function, and species change strategy according to the average payoff of all contributors, but do not depend on the direct payoff of opposite’s gain, $\left[\pi_{i}-\left\langle \pi \right\rangle \right]$.

### 2.2 Non-linear dynamics

To implement bidirectional mutation-based nonlinear dynamics, we use the Fermi pairwise comparison rule in which the intensity of the assessed selection depends on the payoff of opposite neighbors. The replicator equation for this scenario is given by:

(2.1) $${\dot{x}}_{i}=x_{i}\sum_{j\neq i}x_{j}\left(P_{j\leftarrow i}-P_{j\leftarrow i}\right)-\mu x_{i}+\frac{\mu }{n-1}\sum_{j\neq i}x_{i}$$

(2.2) $$P_{i\leftarrow j}=\frac{1}{1+\exp\left[\frac{-\left(\pi_{j}-\pi_{i}\right)}{\kappa }\right]};j\neq i$$

where $P_{i\leftarrow j}$ is a pairwise Fermi function (equation (2.1)) that represents the probability that player i adopts the updating strategy compared to the strategy of j. The parameter κ in equation (2.2) determines the intensity of selection. The payoff difference, $\left(\pi_{j}-\pi_{i}\right)$, where $\pi_{i}$ and $\pi_{j}$ are the fitness of strategy i and j, respectively, is shown in the function’s insets.

### 2.3 Volatile mutation

In contrast to previous works, we introduce the novel idea of volatile mutation by considering the cumulative effect of both cooperators and defectors in an infinitely large and well-mixed population. By considering the impact of a variable mutation rate on the evolution of social behavior in a population, we can gain valuable insights that remain applicable regardless of the specific rates of environmental change and strategy adoption. We can better understand the system's long-term behavior, even as the mutation rate changes on a global scale over time. This allows us to develop a more comprehensive understanding of how social behavior evolves in response to various factors and can inform our understanding of diverse fields, such as biology and sociology. To establish the dynamical equation at the end of each game season, we formulate the mutation dynamics as follows,

(3) $$\frac{d\mu }{d\tau }=\mu \left(1-\mu \right)\left[\theta \sum_{i\neq j}P_{i\leftarrow j}-\left(1-\theta \right)\sum_{i\neq j}P_{j\leftarrow i}\right]$$

In equation (3), μ and (1−μ) characterize the mutation and non-mutation terms, respectively, and θ represents the mutation intensity. Notably, the cumulative probability of both cooperators and defectors is represented by the Fermi pairwise equation $\sum_{i\neq j}P_{j\leftarrow i}$ and $\sum_{i\neq j}P_{i\leftarrow j}$, respectively. The two-stage time systems will affect the mutation process; a single or game season (local time, t) and the mutation evolution adopted at the end of every game season (global time, τ). In this scenario, θ(0≤θ≤1) represents the enhancement fraction only for the cooperators of the resource by the consumer population to alter the resource population. In this scenario, the cumulative cooperators help in augmenting the cooperation while the cumulative defectors act to degrade it. For the time (global), variable μ(τ) increases for θ=1; the system will be akin to a higher presence of a mutation that allows individuals to adopt a new strategy that may be more successful than their current one.

### 2.4 Pairwise 2 × 2 game system

Let us consider a universal dilemma strength-based pairwise game to recognize how volatile mutation for linear and PW-Fermi nonlinear dynamics affect evolutionary game theory. The trivial (TR), prisoner’s dilemma (PD), chicken (CH) and stag-hunt (SH) are the general game classes for exploring the evolution of cooperation in pairwise interaction games given by,

$$\left(\begin{array}{cc} 1 & -D_{r}\\ 1+D_{g} & 0 \end{array}\right)$$

Since, R, S, T and Pare the gaining payoffs of the pairwise game, the GID (Gamble Intended Dilemma) and RAD (Risk Averting dilemma) that have been defined by normalized condition, $D_{r}=P-S$, $D_{g}=T-R$, R=1 and P=0, explore universal dilemma strength [17]. The average payoff of cooperators ($\pi_{C}$) and defectors ($\pi_{D}$) are given by,

(4.1) $$\pi_{C}\left(t\right)=x\left(t\right)-\left(1-x\left(t\right)\right)D_{r}\text{,}$$

(4.2) $$\pi_{D}\left(t\right)=x\left(t\right)\left(1+D_{g}\right)\text{.}$$

Let *x* and ***μ*** represent the relative frequencies of cooperators and mutation, respectively can be written as,

(5) $$\dot{x}\left(t\right)=x\left(t\right)\left(1-x\left(t\right)\right)\left[\pi_{C}\left(t\right)-\pi_{D}\left(t\right)\right]+\mu \left(1-2x\left(t\right)\right)$$

Similarly, for PW-Fermi nonlinear dynamics,

(6) $$\dot{x}\left(t\right)=x\left(t\right)\left(1-x\left(t\right)\right)\left(P_{C}\left(t\right)-P_{D}\left(t\right)\right)+\mu \left(1-2x\left(t\right)\right)$$

The transition probabilities are,

(7.1) $$P_{C}\left(t\right)=\frac{1}{1+\exp\left[\left(\pi_{C}\left(t\right)-\pi_{D}\left(t\right)\right)/\kappa \right]}\text{,}$$

(7.2) $$P_{D}\left(t\right)=\frac{1}{1+\exp\left[\left(\pi_{D}\left(t\right)-\pi_{C}\left(t\right)\right)/\kappa \right]}\text{.}$$

From equations (4.1), (4.2), (5), (6), (7.1), (7.2), at the end of each game season (t→∞), if $P_{C}\left(\infty \right)$ and $P_{D}\left(\infty \right)$ represent the mean payoffs of the cooperator and defector at equilibrium, then the dynamic mutation for the global time scale τ can be written as,

$$\frac{d\mu }{d\tau }=\mu \left(1-\mu \right)\left[\theta P_{C}\left(\infty \right)-\left(1-\theta \right)P_{D}\left(\infty \right)\right]\text{.}$$

In a finite population, we can describe the state of the population based on the number of cooperators, denoted as i. The probability that a cooperator interacts with another cooperator is (i−1)/(N−1), where N is the total population size. The probability that a cooperator will interact with a defector is (N−i)/(N−1). The average payoff for a cooperator, denoted as “$\pi_{C}^{i}$” and a defector as “$\pi_{D}^{i}$”, can be calculated based on these probabilities.

(8.1) $$\pi_{C}^{i}=\frac{i-1}{N-1}-\frac{N-i}{N-1}D_{r}\text{,}$$

(8.2) $$\pi_{D}^{i}=\frac{N-i-1}{N-1}\left(1+D_{g}\right)\text{.}$$

During each step, each individual in the population can update their strategy through a linear or nonlinear imitation process. The overall success of the population, measured by its average payoff, depends on the outcome of these strategy updates. Thus, from equations (8.1), (8.2), we have

$$\left\langle \pi^{i}\right\rangle =\frac{i}{N}\pi_{C}^{i}+\frac{N-i}{N}\pi_{D}^{i}$$

This study examines how mutations impact social cooperation in social networks, focusing on two types of networks: the Random Regular graph and the Barabasi-Albert graph. We analyze how individuals' behavior changes in these networks by varying the mutation rates. When the mutation percentage is set at 0.1 (μ=0.1), approximately 10% of individuals undergo mutation, resulting in a shift in their cooperative or defection strategy. Once an individual undergoes mutation, they do not change their strategy again in subsequent rounds.

## 3 Results and discussion

This section represents the numerical outcomes of the proposed volatile mutation model, considering the linear and non-linear benefit functions in the replicator equations on the evolutionary framework. Additionally, constant and volatile mutations are demonstrated to explain the mutation effect under a pairwise game for both deterministic and stochastic processes. The discussion considers the impact of mutations on dilemma strength and mutation intensity to investigate how different events are influenced by adjusting parameter values.

**Fig. 1:**
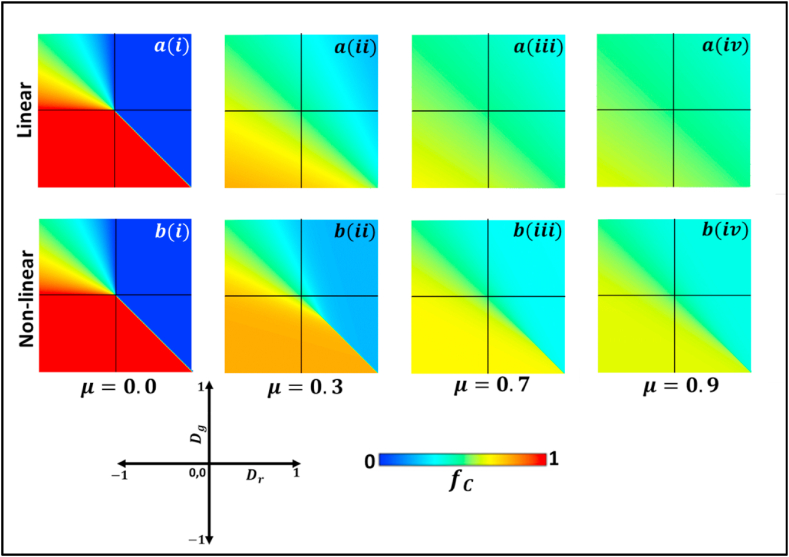
We present the 2D heat diagram for fraction of cooperators $\left(f_{c}\right)$ of the (a) linear and (b) nonlinear (fermi-pairwise) replicator processes for constant mutation aspect in which the mutation rates are (i) μ=0.0 (ii) μ=0.3 (iii) μ=0.7 and (iv) μ=0.9. The red and blue display the fraction of cooperators and defectors individuals, respectively. We observed all four game classes: PD, CH, Trivial, and SH, along with dilemma strength parameters $D_{r}$ and $D_{g}$. It can be seen that an increase in mutation rate increases the fraction of defectors to cooperators and the fraction of cooperators to defectors. (For interpretation of the references to color in this figure legend, the reader is referred to the Web version of this article.)

Fig. 1 depicts the effect of changing the value of μ on linear and nonlinear systems depicted in panel (a) and panel (b), respectively, without granting volatile mutation. From panel (i) to panel (iv), the value of μ is increased gradually increased with μ=0.0,0.3,0.7 and 0.9, while keeping all the other factors constant. The fraction of cooperation, $f_{c}$ measures the level of cooperation in which shades of red represent co-operation, whereas shades of blue represent defection. The value μ=0 gives the basic phase diagram of dynamics classified by $D_{g}$ and $D_{r}$ of two-by-two games [17]. Here, $D_{g}>0$ and $D_{r}>0$ results in the Prisoner’s Dilemma game, $D_{g}<0$ and $D_{r}<0$ in the trivial game, $D_{g}>0$ and $D_{r}<0$ in the chicken game, and, $D_{g}<0$ and $D_{r}>0$ result in the Stag Hunt game. Prisoner’s dilemma (D-dominant) and Trivial (C-dominant) game are colored blue and red, respectively. A gradually shifting fraction of cooperation is observed in the Chicken game region due to the polymorphic phase. Bi-stable shows twofold stages in the Stag Hunt game region; either absorbed all cooperation or all defection.

In the linear system, it can be observed from panels a(i) to a(iv) in Fig. 1 that the level of both cooperation and defection decreases as the value of μ increases in all four regions of the phase diagram, As μ increase further, both cooperation and defection levels reach a level halfway between C and D, represented by cyan color. In this case, all the regions turn cyan, indicating a society where cooperation and defection are identical throughout. In the non-linear system, a similar trend is observed for varying mutations, in which the levels of cooperation and defection decrease with increasing value of μ. However, as each players depends on their opposite neighbors payoff (nonlinearity pairwise comparison), a barrier between cooperation and defection level can be distinguished, unlike in the linear system.

**Fig. 2:**
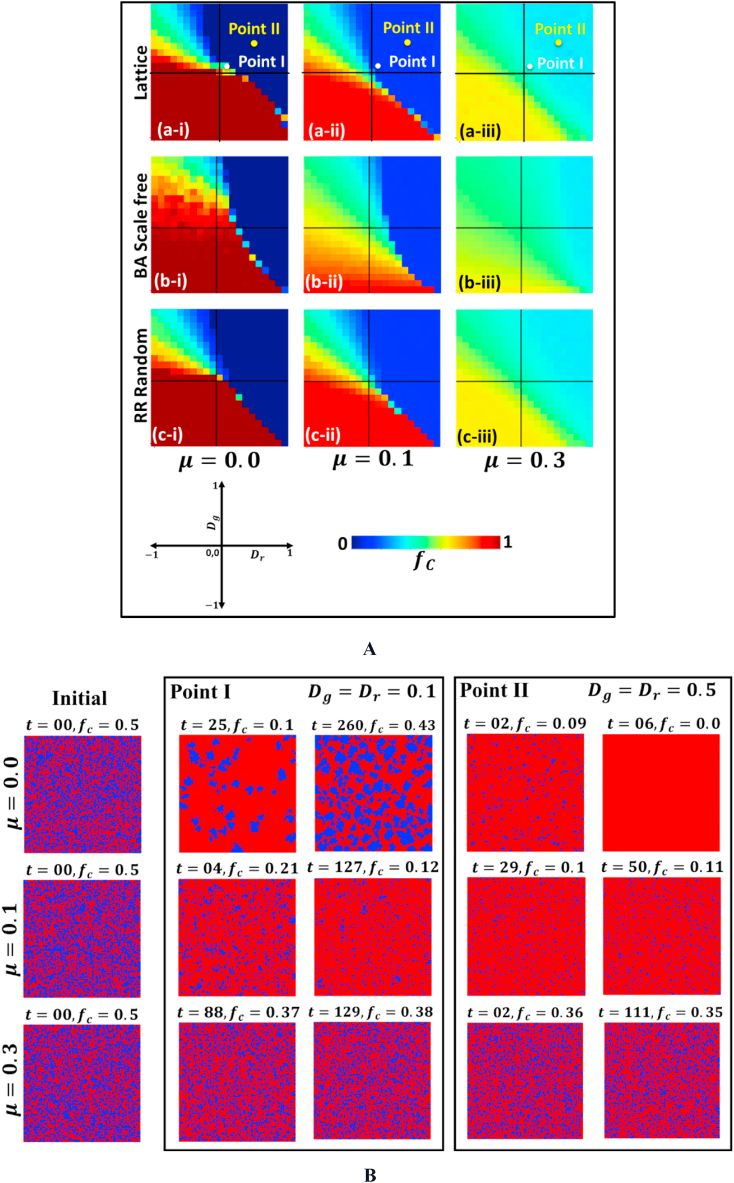
A (Panel A) We present the 2D heat diagram for fraction of cooperators $\left(f_{c}\right)$ of the (a) Lattice, (b) Barbasi-Alberta (BA) scale-free network and (c) RR random network for constant mutation aspect. The red and blue display the fraction of cooperators and defectors individuals. We observed all four game classes: PD, CH, Trivial, and SH, along with dilemma strength parameters $D_{r}$ and $D_{g}$. Here, we consider a reasonable and realistic mutation rate for the MAS approach irrespective of the theoretical approach [50]; the mutation rates are (i) μ=0.0 (ii) μ=0.1 and (iii) μ=0.3. The total population size N = 10,000 and average degree <k>=8. (For interpretation of the references to color in this figure legend, the reader is referred to the Web version of this article.) B. (Panel B) The subsystem presents a stability analysis of two points taken from Fig. 2 (Panel A) for the Prisoner’s Dilemma game at points (i) $D_{g}=D_{r}=0.1$ and (ii) $D_{g}=D_{r}=0.5$. The blue and red colors display cooperator and defector fractions, respectively. The leftmost panel shows the initial setup at t=0. The first, second, and third rows show the square lattice for mutation rate μ=0.0,0.1, and 0.3. Each panel represents for different time and fraction of cooperations $\left(f_{c}\right)$ in both cases distributed uniformly at random. Without mutation (μ=0.0), we obtain the upper right snapshot, where $f_{c}$ is turned to the fully defective situation for Point-II; however, Point-I shows that $f_{c}=0.43$. We obtain that cooperation emerged if we consider the mutation effect μ=0.1 and μ=0.3. (For interpretation of the references to color in this figure legend, the reader is referred to the Web version of this article.)

To emphasize the effect of social networks on pairwise games and mutation, we display Panels (a-i)-(c-iii) in Fig. 2 (A), considering three network approaches (a-*) Latice, (b) Barabasi-Alberta (BA) scale-free network and (c-*) the RR random network. First, we assess a lattice network (a-*) on a pairwise two by two game with mutation used to study how individuals interact in a population and how these interactions can lead to different strategies. The lattice network model assumes that the connections between individuals or entities are limited to their immediate neighbors, creating a grid-like structure that allows for the study of how the local interactions between nodes affect the global behavior of the network. In the presence of mutation, individuals can randomly change their strategy with a certain probability, which introduces variation in the population and can lead to new strategies. The presence of mutation allows individuals to occasionally adopt a new strategy that may be more successful than their current one, even if it is initially rare in the population. As a result, individuals with more successful cooperative strategies can spread through the population, increasing the overall level of cooperation. Secondly, we consider the BA scale-free network (b-*), which has a power-law degree distribution, meaning there are many nodes with low degrees and only a few with very high degrees. For that reason, this can create a situation where the number of cooperators rapidly increases among the low-degree nodes, but the cooperation takes longer to reach the high-degree hubs sub-panel (a–i). In pairwise games, two players interact with each other and receive a payoff based on their chosen strategies. The game's outcome can affect the players' fitness, which in turn affects their chances of reproducing and passing on their traits to the next generation. Mutation is a natural mechanism that can introduce new strategies into a population, leading to the emergence of novel behaviors and the evolution of existing ones. In pairwise games, mutation can lead to the creation of new strategies or the elimination of existing ones, and its effect on the game’s evolution can depend on the network’s structure. It can be observed that mutation can promote the evolution of cooperation in the Prisoner’s Dilemma game, especially in networks with a high degree of heterogeneity [44,45]. However, sub-panel(c-*) presents slower emergence of cooperation for RR random regular networks due to their moderate heterogeneity nature. The presence of mutation can enhance the evolution of cooperation in the Public Goods game on RR random networks; mutation can help to overcome the problem of the low clustering coefficient of RR random networks and promote the emergence of cooperators [46,47].

In the Prisoner's Dilemma game context, the Lattice snapshot as a subsystem conducted a stability analysis of two points from Fig. 2 (Panel B) [48]. The analysis focused on two specific points, (I) $D_{g}=D_{r}=0.1$ and (II) $D_{g}=D_{r}=0.5$ and used the blue and red colors to represent the cooperator and defector fractions, respectively. The leftmost panel in the figure showed the initial setup at t=0, while the first, second, and third rows showed the square lattice for mutation rate μ=0.0, 0.1, and 0.3, respectively. The panels represented different times and fractions of cooperations ($f_{c}$) in both cases that randomly distributed. Without no impact of mutation, the upper right snapshot showed that the $f_{c}$ turned to the fully defective situation for Point II. However, Due to network reciprocity in Point-I, the $f_{c}$ is 0.43, indicating the emergence of cooperation. The result for the case of μ=0.1 and μ=0.3 showed that cooperation emerged in both cases, indicating that mutation significantly impacted the outcome of the Prisoner's Dilemma game. The findings of this analysis highlight the importance of considering mutation in the context of game theory and evolutionary dynamics to enhance cooperation.

**Fig. 3:**
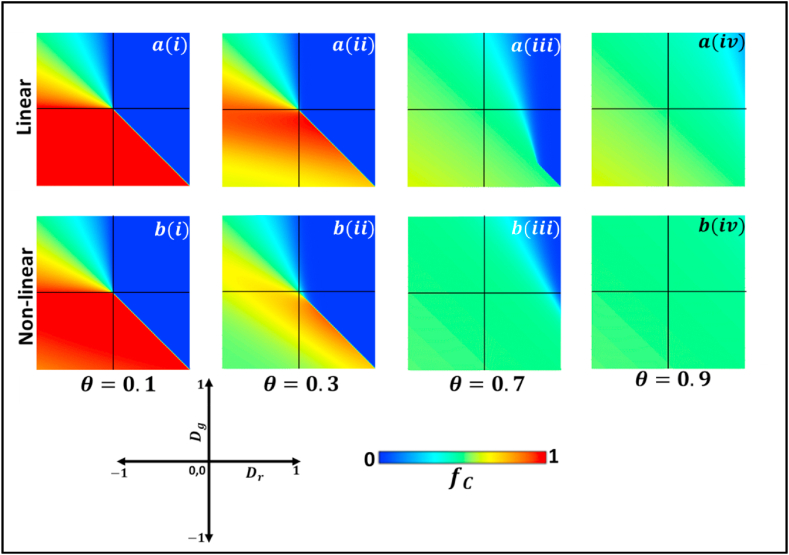
We present the 2D heat diagram for fraction of cooperators $\left(f_{c}\right)$ of the (a) linear and (b) nonlinear (fermi-pairwise) replicator processes for dynamic mutation aspect in which the cooperation enhancement rates are (i) θ=0.0 (ii) θ=0.3 (iii) θ=0.7 and (iv) θ=0.9. The red and blue display the fraction of cooperators and defectors individuals. We observed all four game classes: PD, CH, Trivial, and SH, along with dilemma strength parameters $D_{r}$ and $D_{g}$. For lower θ<0.5, the fraction of cooperation reduced (Trivial), and for higher θ>0.5, the fraction of cooperation increased (PD). (For interpretation of the references to color in this figure legend, the reader is referred to the Web version of this article.)

Fig. 3 illustrates how the increase in the value of mutation intensity, θ affects linear and non-linear systems with volatile mutation. Although, Fig. 3 shows a similar tendency to Fig. 1, in Fig. 3 from panel (i) to panel (iv), the value of θ is gradually increased, while keeping all the other factors constant. At θ=0.1, the primary 2D phase diagram of the model, classified by $D_{g}$ and $D_{r}$ of two-by-two game, is obtained for both linear and non-linear systems. However, a closer look shows that the cooperation level in the non-linear system is slightly diminishing, represented by orange color. Our model setup shows that the mutation intensity (θ) controls the mutation rate based on the collective cooperation or defection level. Therefore, when θ=0.3, defection is more substantial, which is reflected in the phase diagram where the red region, representing cooperation, is diminishing in both linear and non-linear systems. The blue area slowly fades as it increases further, approaching a more cooperative society in both linear and non-linear systems. However, a non-linear system tends towards a more unified society compared to a linear system.

**Fig. 4:**
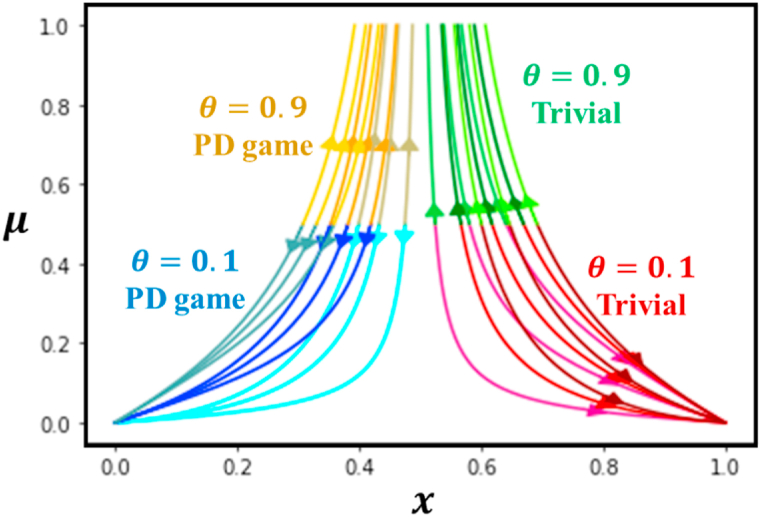
Phase trajectory line graphs on x−μ plane (cooperators and mutation) under the influence of evolutionary game theory and mutation game dynamics. The line-colored green, yellow, blue, and red represent Trivial with θ=0.9, PD with θ=0.9, PD with θ=0.1, and Trivial with θ=0.1, respectively. Inside this, we consider various settings for $D_{g}$ and $D_{r}$ as (0.1, 0.1), (0.1,0.5), (0.1,0.9), (0.5, 0.1), (0.5,0.5), (0.5,0.9), (0.9, 0.1), (0.9,0.5), and (0.9,0.9). Here, for lower θ(=0.1), the individuals the system converges to μ→0, meaning irrespective of mutation. On the other hand, for large θ(=0.9), the mutation rate approaches 1 (μ→1). (For interpretation of the references to color in this figure legend, the reader is referred to the Web version of this article.)

According to the above discussion, we have only examined the impact of a fixed mutation rate on cooperative behavior, which assessed whether individuals' cooperation changed with a constant mutation rate. However, we did not explore the possibility of individuals having a volatile mutation that can adapt to environmental feedback and evolve their strategies accordingly. To investigate this, we now focus on dynamic time-dependent mutation dynamics within the framework of EGT (Evolutionary Game Theory). Fig. 4 displays the trajectory time series line of x, the time series of μ, and the phase trajectory projected on the x−μ plane to how an initial setting (x,μ)=(0.5,0.5) approaches 0 or 1.

There are four possibilities that correspond to different combinations of the relative values of θ=0.1 and θ=0.9 for the Trivial game ($D_{g}<0$ and $D_{r}<0$) and PD game ($D_{g}>0$ and $D_{g}>0$). When θ is lower (θ=0.1), and individuals play a trivial game, the system converges to (x,μ)→(1,0). On the other hand, when θ is lower (θ=0.1), and individuals play the PD game, the system converges to (x,μ)→(0,0). The opposite situation occurs when large θ values are presumed. As a result, if θ(=0.9) is higher, the mutation rate (μ) approaches 1 even if the cooperation fraction is intermediate. Thus, the fraction of cooperation will approach an intermediate value (x→0.5) for higher values of θ, regardless of whether individuals choose a cooperation or defection strategy.

**Fig. 5:**
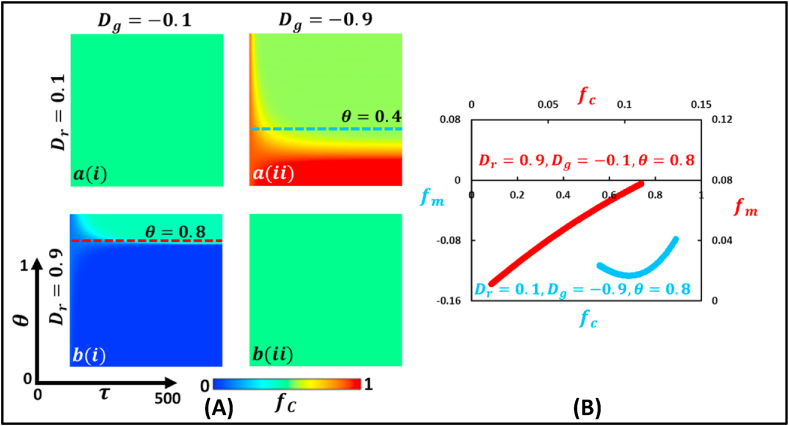
Phase diagram of fraction of cooperation illustrates the Chicken type game, where $D_{r}>0$ and $D_{g}<0$. The left panel (Panel A) demonstrates a 2D heat diagram that depicts the effect of changing mutation intensity, θ, and generation. The right panel (Panel B) present a generation trajectory graph that shows the fraction of mutated individuals, $f_{m}$, concerning the change in the fraction of cooperative individuals, $f_{c}$ (blue and red dotted line) for θ=0.4 and θ=0.8. (For interpretation of the references to color in this figure legend, the reader is referred to the Web version of this article.)

**Fig. 6:**
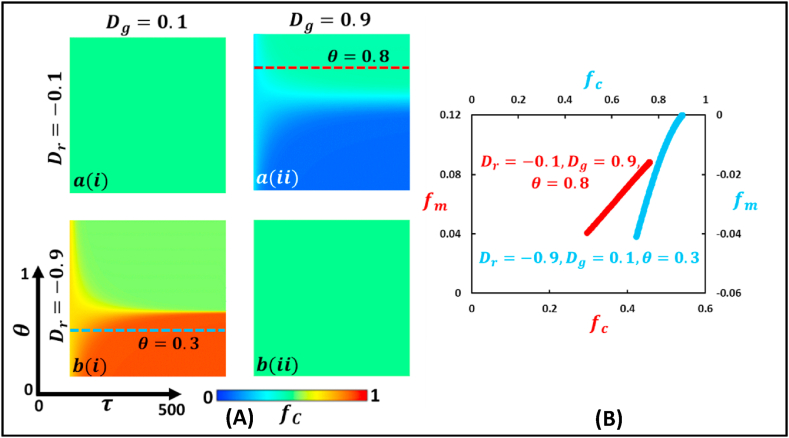
Phase diagram of fraction of cooperation illustrates the stag hunt type game, where $D_{r}<0$ and $D_{g}>0$. The left panel (Panel A) demonstrates a 2D heat diagram that depicts the effect of changing mutation intensity, θ, and generation. The right panel (Panel B) present a generation trajectory graph that shows the fraction of mutated individuals, $f_{m}$, concerning the change in the fraction of cooperative individuals, $f_{c}$ (blue and red dotted line) for θ=0.3 and θ=0.8. (For interpretation of the references to color in this figure legend, the reader is referred to the Web version of this article.)

**Fig. 7:**
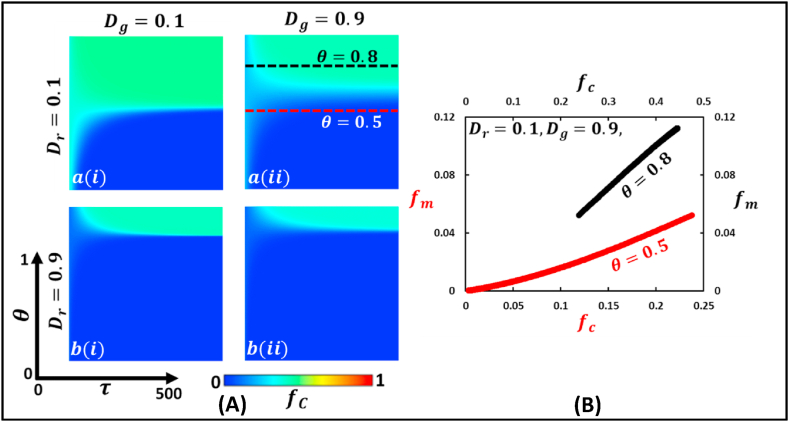
Phase diagram of fraction of cooperation illustrates the Prisoner’s dilemma type game, where $D_{r}>0$ and $D_{g}>0$. The left panel (Panel A) demonstrates a 2D heat diagram that depicts the effect of changing mutation intensity, θ, and generation. The right panel (Panel B) present a generation trajectory graph that shows the fraction of mutated individuals, $f_{m}$, concerning the change in the fraction of cooperative individuals, $f_{c}$ (black and red dotted line) for θ=0.8 and θ=0.5. (For interpretation of the references to color in this figure legend, the reader is referred to the Web version of this article.)

**Fig. 8:**
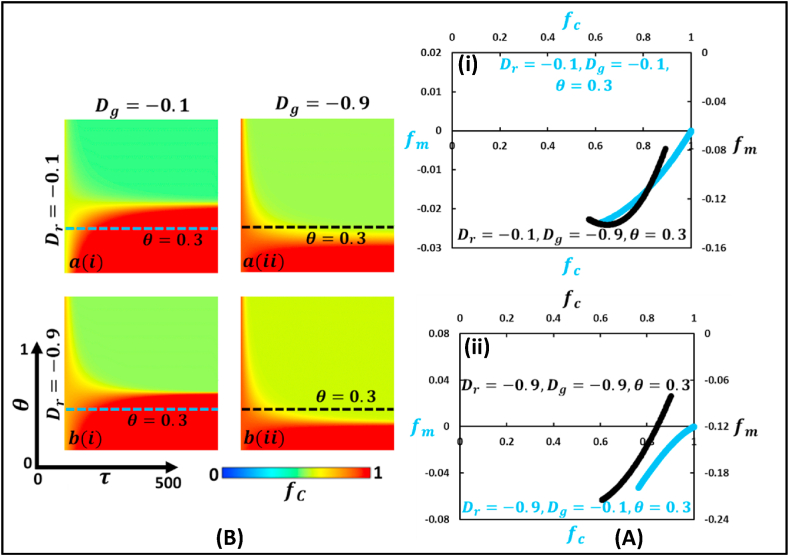
Phase diagram of fraction of cooperation illustrates the Trivial type of game, where $D_{r}<0$ and $D_{g}<0$. The left panel (Panel A) demonstrates a 2D heat diagram that depicts the effect of changing mutation intensity, θ, and generation. The right panel (Panel B) present a generation trajectory graph that shows the fraction of mutated individuals, $f_{m}$, concerning the change in the fraction of cooperative individuals, $f_{c}$; (i) black and blue dotted line for θ=0.3 and (ii) black and blue dotted line for θ=0.3. (For interpretation of the references to color in this figure legend, the reader is referred to the Web version of this article.)

The next step is to provide the 2D heat diagram (panel A) and trajectory plot (panel B) of four types of games (Fig. 5, Fig. 6, Fig. 7, Fig. 8) that depict the effect of changing mutation intensity, θ, and generation, γ. For this purpose, Fig. 5 (Panel A and Panel B) illustrates the chicken type game, where $D_{r}>0$ and $D_{g}<0$. The left panel (Panel A) demonstrates a 2D heat diagram that depicts the effect of changing mutation intensity, θ, and generation, γ, on CH. On the other hand, the right panel (Panel B) of Fig. 5 is a generation trajectory graph that displays the fraction of mutated individuals, $f_{m}$, concerning the change in the fraction of cooperative individuals, $f_{c}$ by referring right panel (blue and red dotted line).

At block a(i) and block b(ii), where $D_{r}=0.1$, $D_{g}=-0.1$ and $D_{r}=0.9$, $D_{g}=-0.9$, respectively, the fraction of individuals in a society mutates and reaches an equilibrium where it is no longer purely cooperative or purely defective but instead a point halfway between cooperation and defection, represented by the cyan color.

Meanwhile, in block a(ii) ($D_{r}=0.1$ and $D_{g}=-0.9$), when both τ and θ(<0.3) are comparatively small, a higher fraction of cooperation is observed, represented by the red color. However, for θ(>0.3) and higher τ, there is no effect of cooperation and defection on the society, which remains halfway between cooperation and defection. Finally, in block b(i) $(D_{r}=0.9$ and $D_{r}=-0.1)$, when the generation number is lower, the society remains defective, represented by the blue color, except for higher θ. However, for higher θ(<0.8), as the generation τ increases, the inadequate tendency diminishes and reaches a level halfway between cooperation and defection, represented by the cyan color.

Besides, in the right panel (B), the red curve represents the trajectory for $D_{r}=0.9$ and $D_{g}=-0.1$ at θ=0.8, which shows an increasing tendency (a straight line), indicating that the fraction of mutated individuals, $f_{m}$ increases as the fraction of cooperative individuals, $f_{c}$, expands. On the other hand, the blue curve represents the trajectory for $D_{r}=0.1$ and $D_{g}=-0.9$, showing a parabola where the fraction of mutated individuals, $f_{m}$, decreases with an increase in the fraction of cooperative individuals, $f_{c}\text{,}$ up to a certain point, after which it increases with a further increase in $f_{c}$.

Fig. 6 (Panel A and Panel B) illustrates the stag hunt type game with the same settings as the Chicken type game (Fig. 5), but with opposite settings for the dilemma strength, $D_{r}<0$ and $D_{g}>0$. We can see from block a(i) and block b(ii) that the fraction of individuals in a society mutates and reaches an equilibrium that always stays between the pure cooperative and pure defective regions, colored with cyan. Meanwhile, in block a(ii) $(D_{r}=-.01$ and $D_{r}=0.9)$, when the generation number is less, the society remains defective, represented by the blue color, except for higher θ. However, for higher θ(>0.8), as the generation τ increases, the inadequate tendency diminishes and reaches a halfway level. Finally, at block b(i) ($D_{r}=-0.9$ and $D_{g}=0.1$), when both τ and θ(<0.3) are comparatively small, a higher fraction of cooperation is observed, represented by the red color. However, for θ(>0.3) and higher τ, there is no effect of cooperation and defection on the society. Let us now turn our attention to the right panel (B), where the red curve represents the trajectory for $D_{r}=-0.1$ and $D_{g}=0.9$ at θ=0.8, which presents an increasing tendency (straight line), meaning the fraction of mutated individuals, $f_{m}$ increases with an expanding fraction of cooperative individuals, $f_{c}$. On the other hand, the blue curve represents the trajectory for $D_{r}=-0.9$ and $D_{g}=0.1$, and presents a parabolic curve here an increase in the fraction of cooperative individuals, $f_{c}\text{,}$ initially leads to a decline in the portion of mutated individuals, $f_{m}\text{,}$ up to a certain point, and then it increases with a further increase in $f_{c}$.

It has already been well-defined that the stability behavior of the PD game is likewise a defective game. If we consider panel A in Fig. 7 for the PD game and vary along θ and τ, some enhancements of the fraction of cooperation have been observed. In all blocks, for lower values of θ and slightly higher τ values, the society remains in a defective situation, represented by the blue color. However, increasing the θ values enhance the fraction of cooperation; the species mutates and reaches an equilibrium that is neither cooperative nor defective, represented by the cyan color. However, a higher value of θ, τ does not affect the stability. By deliberately observing all blocks, if we consider a reasonably lower dilemma strength (block a(i)), the heat map shows a higher cooperation fraction because of randomness coming from the mutation noise effect. Furthermore, in the right panel 7 (B), both the red and black trajectory curves shows that the fraction of mutated individuals, $f_{m}$ increases (straight line) with an expanding fraction of cooperative individuals, $f_{c}$.

Finally, Fig. 8 (Panel A and Panel B) displays the trivial type of game, where $D_{r}<0$ and $D_{g}<0$. As for comparatively lower θ values, the society remains unchanged, which is cooperative. However, as θ increases, the individuals mutate and reach a halfway equilibrium between cooperation and defection. Furthermore, the blue curve in the right panel (B) shows a similar increasing tendency; the fraction of mutated individuals, $f_{m}$ increases with an expanding fraction of cooperative individuals, $f_{c}$. However, the black curve represents the trajectory for $D_{r}=-0.1$ and $D_{g}=-0.9$ at θ=0.3, which is a parabola where an increase in the fraction of cooperative individuals, $f_{m}$, causes the fraction of mutated individuals, $f_{c}$ to decreases up to a certain point and then increase.

While networks have been useful in studying the impact of mutations on social cooperation in EGT, there are still some limitations to consider. One limitation is that the current network work is relatively simple and does not consider dynamic mutation for network topologies. Thus, the results obtained from networks may only partially reflect the real-world complexities of social networks that will be considered in our future works.

## 3 Conclusion

In this work, we have considered two aspects-linear and nonlinear dynamics in evolutionary game theory and how they are affected by volatile mutation. We have shown and explained 2D heat diagrams of the linear and PW-Fermi nonlinear replicator dynamics for constant mutation aspects by varying mutation rates, μ, and mutation intensity rates, θ. Then we have shown and explained phase diagrams of the fraction of cooperation that illustrate the Chicken, Stag Hunt, Prisoner’s Dilemma and Trivial type games for both deterministic and stochastic aspects. Additionally, we have shown: i) 2D heat diagrams that depict the effect of changing mutation intensity, θ, and generation, and ii) generation trajectory graphs that shows the fraction of mutated individuals, $f_{m}$, concerning the change in the fraction of cooperative individuals, $f_{c}$.

The impact of mutation on the complexity and biodiversity of evolutionary processes has long been recognized as a significant aspect of human behavior and biological contexts. Understanding how mutation affects uncertain social dilemmas, such as coordination or cooperation dilemmas, is a fundamental question in evolutionary dynamics. In our study, we have addressed this question by explicitly incorporating volatile mutation in the analysis of natural games. Our findings provide compelling evidence of how mutation influences game classes, including Stag Hunt (SH), Prisoner's Dilemma (PD), Chicken (CH), and Trivial games, highlighting its notable role in shaping their dynamics. In a game context, our results indicate that the cooperative payoff of the population increases if players mutate with higher mutation intensity. Furthermore, mutation can also mean randomized exploration: people explore the strategy space by experimenting with new strategies. In sum, a high mutation probability seems to be appropriate for social evolutionary dynamics. Our conditions can be applied to the initial analysis of any evolutionary game specified by an n×n payoff.

Irrespective of non-constant mutation [49], the presence of mutations can significantly impact the game's outcome in both types of networks. In the case of a Barabasi-Albert network, a higher mutation rate can result in more cooperation among the hubs, which can lead to the adoption of cooperative strategies. However, in a Random Regular network, the presence of mutations can decrease the level of cooperation. Because individuals in this type of network are more likely to interact with their neighbors, mutations that result in defection can spread more easily. Overall, these findings suggest that the network's structure can significantly impact the outcome of an evolutionary game, particularly when mutations are present.

## Funding statement

This research did not receive any specific grant from funding agencies in the public, commercial, or not-for-profit sectors.

## Author contribution statement

K M Ariful Kabir: Conceived and designed the experiments; Performed the experiments; Analyzed and interpreted the data; Contributed reagents, materials, analysis tools or data; Wrote the paper.

MD Shahidul Islam: Performed the experiments; Analyzed and interpreted the data; Contributed reagents, materials, analysis tools or data.

Sabawatara Nijhum: Analyzed and interpreted the data; Wrote the paper.

## Data availability statement

No data was used for the research described in the article.

## Declaration of competing interest

The authors declare that they have no known competing financial interests or personal relationships that could have appeared to influence the work reported in this paper
